# Optical information processing using dual state quantum dot lasers: complexity through simplicity

**DOI:** 10.1038/s41377-021-00670-y

**Published:** 2021-11-29

**Authors:** Bryan Kelleher, Michael Dillane, Evgeny A. Viktorov

**Affiliations:** 1 Department of Physics, University College Cork, Cork, Ireland; 2 Tyndall National Institute, University College Cork, Lee Maltings, Dyke Parade, Cork T12 R5CP Ireland; 3 Centre for Advanced Photonics & Process Analysis, Munster Technological University, Bishopstown, Cork T12 P928 Ireland; 4 National Research University of Information Technologies, Mechanics and Optics, Kronverksky Pr. 49, St. Petersburg, 197101 Russia

**Keywords:** Semiconductor lasers, Quantum dots

## Abstract

We review results on the optical injection of dual state InAs quantum dot-based semiconductor lasers. The two states in question are the so-called ground state and first excited state of the laser. This ability to lase from two different energy states is unique amongst semiconductor lasers and in combination with the high, intrinsic relaxation oscillation damping of the material and the novel, inherent cascade like carrier relaxation process, endows optically injected dual state quantum dot lasers with many unique dynamical properties. Particular attention is paid to fast state switching, antiphase excitability, novel information processing techniques and optothermally induced neuronal phenomena. We compare and contrast some of the physical properties of the system with other optically injected two state devices such as vertical cavity surface emitting lasers and ring lasers. Finally, we offer an outlook on the use of quantum dot material in photonic integrated circuits.

## Introduction

Quantum confinement of carriers in semiconductor material is responsible for many of the technological advances in modern technology, being central to the vast majority of semiconductor lasers in use worldwide. Quantum well based devices are dominant but there are many reasons to believe that going beyond the one dimensional confinement could lead to technological advantages. Quantum dot (QD) semiconductor material offers the ultimate possible quantum confinement, with dimensions on the order of the de Broglie wavelength in all three spatial dimensions^1^. Lasers that use InAs based QDs as the active material have many different properties to conventional semiconductor lasers and display unique dynamics when in coupled configurations or when undergoing feedback. When free-running, they offer advantages over conventional devices, with low pump threshold values^2–4^ and excellent temperature insensitivity^5,6^. They also display an unusually high damping of the relaxation oscillations (ROs), one of the most important dynamical characteristic of these devices^7,8^. In contrast, the RO damping in conventional semiconductor lasers is very weakly damped. The high damping of InAs QDs is a consequence of the carrier occupation of the material. The dot occupation probability is high and so there is little scope for oscillations in the carriers and relaxation oscillations are quickly damped out. This high damping in turn has many consequences for their behaviour in unidirectional and bidirectional optical injection configurations and when undergoing external optical feedback. These devices are extremely stable in feedback configurations^8^ and offer genuine promise of isolator free operation^9–11^. In the optical injection configuration they are significantly more stable than conventional semiconductor lasers, allowing for phase locked operation over a much larger area of control parameters and with a marked reduction in areas displaying chaotic operation^12–17^. They also display many unique dynamics such as canard phenomena and a multitude of excitable regimes^16–22^. The RO stability means that they are also extremely stable when mutually coupled, even for long delays between the devices^23^.

For electrically pumped quantum well based lasers, even with the partially discretised density of states, the lasing emission is always at the frequency of the lowest energy transition (typically called the ground state, GS). This is not the case with InAs QD based lasers. One can saturate the GS in such devices and lasing can be obtained from higher energy transitions - the so-called excited states (ESs)^24^. In particular, lasing from the first ES is regularly obtained. By continuously increasing the electrical pumping of such a QD laser, a typical evolution is to obtain a threshold for GS emission followed by a second threshold where ES emission arises and simultaneous GS and ES lasing is obtained, and finally, quenching of the GS emission after which ES emission only is obtained^25–31^. In fact, for very short devices or where losses are high, GS emission can be entirely removed, and ES emission only obtained^32^. This dual state nature opens up the possibility for many unique optical injection scenarios, of interest for fundamental science and for technological applications; chief among these are bistabilities with switching mechanisms and neuromorphic functionality. We focus on these below and also look forward to exciting integration developments and photonic integrated circuits based on QD lasers^33^.

Bistabilities and switching mechanisms in semiconductor lasers are topics of great interest both fundamentally and for applications including signal processing, optical flip-flops, optical memory elements and optical logic gates^34–39^. A particularly successful technique for obtaining bistable operation is via the optical injection of devices that admit two lasing states such as VCSELs^40–46^, semiconductor microring lasers^47,48^, photonic crystal lasers^49^ and two-colour Fabry-Pérot lasers^50,51^. The two states in VCSELs arise from two polarisation states, naturally arising due to the broad area of the emitting surface in such devices. In semiconductor microring lasers, light can propagate either in the clockwise direction or in the anticlockwise direction, leading to two different directional states in this case, again arising naturally from the geometry of the system. On the other hand, the two-colour Fabry-Pérot devices are explicitly designed to emit from two distinct longitudinal modes via careful design of a pattern of slots etched into the active material. When undergoing optical injection, all of such devices can be used as optical switches and all-optical memory elements. The dual state possibilities of QD lasers means they are also of interest for such applications. However, the carrier dynamics in QD devices are very different from all of these other cases. There is a cascade like relaxation pathway for carriers in QD material, from the carrier reservoir, through the ES and on to the ground state^26,27,31^. Superficially, there are similarities with the other dual emission devices but the physical underpinnings are quite different and allow for phenomena that cannot be obtained with conventional semiconductor lasers as described below. Even with QD based devices such as QD VCSELs, both modes emit from the GS and so the cascade relaxation does not have the influence it does in our ES-GS case. What’s more, edge emitting QD lasers rely on relatively simple fabrication and their geometry is ideally-suited to in-plane integration.

Apart from injection^52,53^, researchers have considered dual state optical feedback configurations^54–64^ where competition between states and the states’ different RO characteristics are often central to results; dual state mode-locking where resistor self-electrooptic-effect device configurations and neuronal applications have been considered among others^65–73^; and modulation characteristics when emitting from the ES^74–79^, where it is shown that emission from the ES can significantly improve the modulation properties of QD lasers. We do not delve into these results here but instead restrict ourselves to optical injection.

## Bistability and state switching

**Fig. 1:**
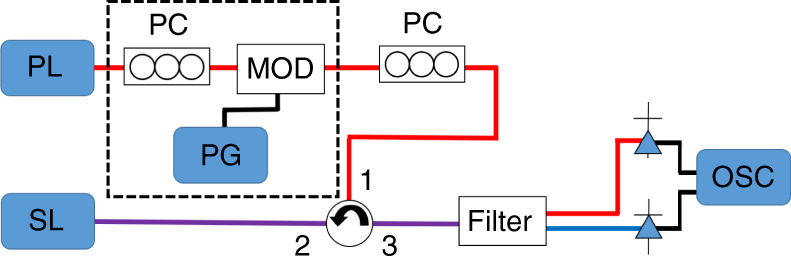
Typical optical injection set up used in several experiments described below. The most basic setup can be built without the apparatus in the dashed box. Light from a primary laser (PL), typically a tunable laser source (TLS) goes through a circulator (providing optical isolation of ~ 40 dB) and is injected into the cavity of the secondary laser (SL), the QD laser. A polarisation controller (PC) maximises coupling. A filter is used to separate the ground state (GS) and excited state (ES), so each state can be monitored independently by detectors connected to an oscilloscope (OSC). To modulate the injected light additional apparatus is included, shown in the box with dashed edges. A polarisation controller (PC) is used to send light with the correct polarisation into a phase modulator (MOD) to trigger excitable pulses^32^ or Mach Zender modulator^80^ to induce switching. The modulator is driven with a pulse generator (PG)

For most of the work described here, we consider the optical injection of a dual state QD laser biased so as to emit from the ES only, while the optical injection is at the GS frequency. Figure 1 shows the basic concept of an optical injection experiment on a dual state laser. All experiments discussed below will be based on this core setup. Light is coupled from the tunable, primary laser (PL) through port 1 and out of port 2 of an optical circulator providing ~ 40 dB optical isolation, to a polarisation controller (PC) and into the secondary laser (SL), the QD device. The light from the SL then passes into port 2 of the circulator and out of port 3, after which it is filtered so that the GS and ES can be detected and analysed independently.

**Fig. 2:**
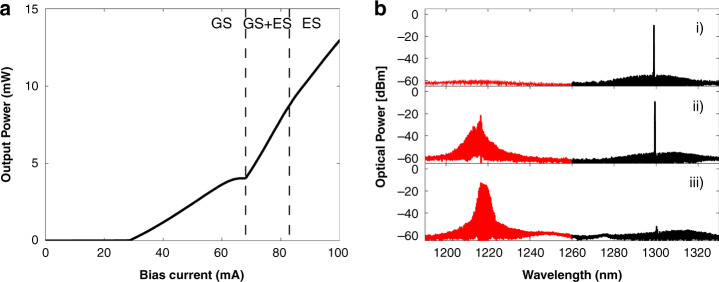
**a** LI curve of the two colour device used in that demonstrates the typical features observed in most devices discussed in this review. **b** Emission spectra of the device corresponding to 3 distinct lasing regions: (i) GS only emission following the GS threshold; (ii) simultaneous GS and ES emission following the ES threshold; (iii) ES only emission following quenching of the GS. Reprinted with permission from^80^^©^The Optical Society

**Fig. 3:**
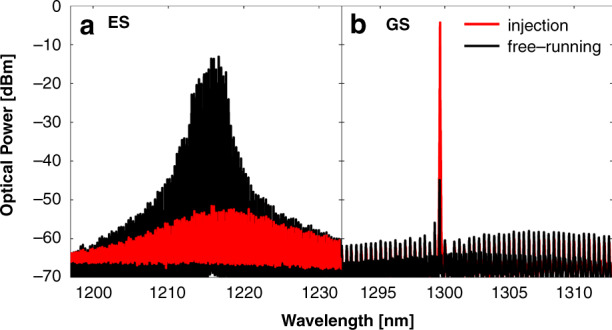
**a** ES spectra and **b** GS spectra before and after optical injection into the GS. Initially the device is pumped so as to emit from the ES only and all the GS modes are subthreshold (black). Injecting close to a GS mode the ES turns off and the GS turns on (red). Reprinted with permission from^80^^©^The Optical Society

In^80^ a QD device fabricated to lase from a single longitudinal mode in the GS while remaining multimode when operating from the ES is experimentally analysed. In the free-running configuration the first threshold is for GS only lasing, after which simultaneous GS and ES lasing is obtained and finally, ES only lasing as shown in Fig. 2. In^80^ it is electrically pumped so as to emit from the ES only. Light from a primary laser, in this case a tunable laser source (TLS) emitting near the frequency of the GS, is injected into the QD secondary laser. For sufficiently low detuning (the frequency of the primary laser minus that of the secondary laser), the ES is completely suppressed and light is emitted from the GS only, see Fig. 3. As pointed out in^81^, when the QD laser emits from only the ES, all GS modes are subthreshold. This makes the experimental determination of the the detuning difficult. Thus, the frequency of the primary laser is instead pragmatically set to where a desired qualitative behaviour is achieved in the analysis of the different dynamical regimes. examined.

**Fig. 4:**
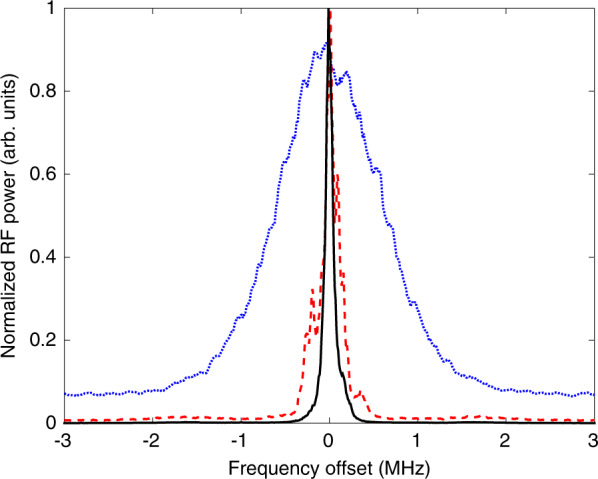
Three linewidth measurements. The blue line shows the linewidth of the GS when the laser is pumped to emit from GS only. The black line shows the narrow linewidth of the primary laser, the TLS. The dashed red line shows the linewidth of the QD laser after injection. Phase locking is verified as the QD laser attains the linewidth of the primary laser. Reprinted with permission from^81^^©^The Optical Society

To confirm that the emission is phase locked to the injected light, the linewidth is investigated using a delayed self-heterodyne experiment with the results as shown in Fig. 4 from^81^. Before any injection, the linewidths of the primary and secondary lasers are found separately. When free-running and emitting from the GS only, the linewidth of the QD laser is ~1 MHz. The linewidth of the tunable laser is resolution limited, but <100 kHz. The current applied to the QD laser is then increased until it lases from the ES only. With optical injection the ES can be quenched and GS emission recovered. The linewidth of the QD laser is then found to be less than 100 kHz, confirming the phase-locking of the QD laser to the tunable laser. The frequency of the PL is now set to where the highest GS output of the QD laser is achieved and the injection strength is swept up and down. While there are instances of oscillations noted, dynamics are not investigated in^81^. Instead, only the average output powers of the GS and ES are reported. A hysteresis loop is found indicating a bistability. Both states in this bistability display simultaneous ES and GS lasing; the bistability is between a state where the GS dominates the emission and one where the ES dominates. The findings are investigated using a rate equation model designed for the dual state QD system. This model consists of rate equations for the GS field, the ES intensity, the occupation probabilities for holes and electrons in the ES and in the GS and the carrier population in the carrier reservoir.

1 $$\begin{array}{l}{\dot{E}}_{g}=\frac{1}{2}\left[(1+i\alpha )(2{g}_{0}^{g}({n}_{e}^{g}+{n}_{h}^{g}-1)-1)\right.\\ \qquad\;\left.+\,i4\beta {g}_{0}^{e}({n}_{e}^{e}+{n}_{h}^{e}-1)\right]{E}_{g}-i{E}_{g}{{\Delta }}+\varepsilon \end{array}$$

2 $${\dot{I}}_{e}=[4{g}_{0}^{e}({n}_{e}^{e}+{n}_{h}^{e}-1)-1]{I}_{e}$$

3 $$\begin{array}{ll}{\dot{n}}_{e,h}^{g}&=\eta \left[2{B}_{e,h}{n}_{e,h}^{e}(1-{n}_{e,h}^{g})-2{C}_{e,h}{n}_{e,h}^{g}(1-{n}_{e,h}^{e})\right.\\ &\left.-\,{n}_{e}^{g}{n}_{h}^{g}-{g}_{0}^{g}({n}_{e}^{g}+{n}_{h}^{g}-1){\left|{E}_{g}\right|}^{2}\right]\end{array}$$

4 $$\begin{array}{ll}{\dot{n}}_{e,h}^{e}&=\eta \left[-{B}_{e,h}{n}_{e,h}^{e}(1-{n}_{e,h}^{g})+{C}_{e,h}{n}_{e,h}^{g}(1-{n}_{e,h}^{e})\right.\\ &+\,{B}_{e,h}^{w}{n}_{e,h}^{w}(1-{n}_{e,h}^{e})-{C}_{e,h}^{w}{n}_{e,h}^{e}-{n}_{e}^{e}{n}_{h}^{e}\\ &\left.-\,{g}_{0}^{e}({n}_{e}^{e}+{n}_{h}^{e}-1){I}_{e}\right]\end{array}$$

5 $$\begin{array}{ll}{\dot{n}}_{e,h}^{w}&=\eta \left[J-{n}_{e}^{w}{n}_{h}^{w}-4{B}_{e,h}^{w}{n}_{e,h}^{w}(1-{n}_{e,h}^{e})\right.\\ &\left.+\,4{C}_{e,h}^{w}{n}_{e,h}^{e}\right]\end{array}$$

A dot denotes differentiation with respect to $t=\tilde{t}/{\tau }_{ph}$, where $\tilde{t}$ is time and *τ*_*ph*_ is the photon lifetime. *η* = *τ*_*ph*_/*τ*, where *τ* denotes the carrier recombination time. The subscripts *e* and *h* represent electron and hole, respectively. *α* is the usual GS phase-amplitude coupling. For simplicity this is taken to be a constant in^81^. Despite this simplification, this assumption is sufficient to reproduce the experimental findings. ${g}_{0}^{g}$ and ${g}_{0}^{e}$ are gain coefficients. The control parameters are the injection strength *ε*, and the detuning between the frequency of the injected light and that of the GS, Δ ≡ *ω*_*i*_ − *ω*_*g*_. *J* is the pump current. *B*_*e*,*h*_ and ${B}_{e,h}^{w}$ are the normalised capture rates to the GS and ES, respectively, while the *C* terms are the normalised escape rates and are linked to the capture rates via Kramers type relations, as described in^81^. The different spin degeneracies in the QD energy levels, Pauli blocking, interstate captures and escapes are all included.

**Fig. 5:**
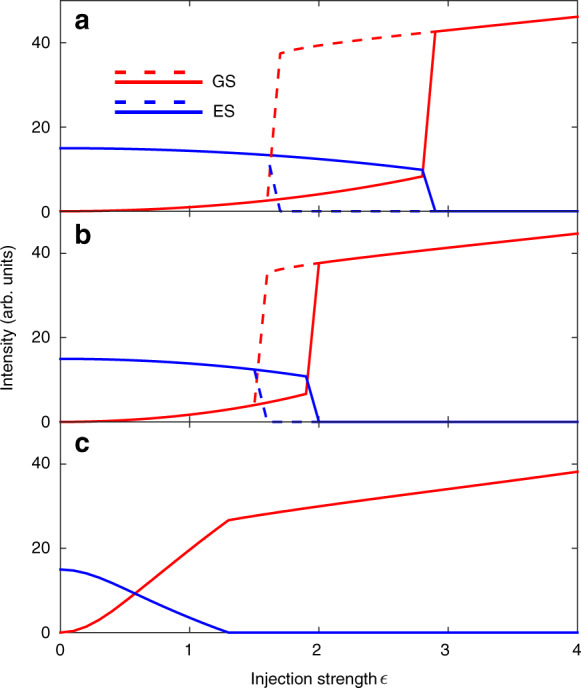
Hysteresis cycle analysis via equations (1)-(5). The QD laser is biased to emit from the ES when free-running. The panels show the effect of changing the *β*-factor. **a** *β* = 3, (**b**) *β* = 2.5, and (**c**) *β* = 1. For *β* > 2 the experimentally observed bistabilty is reproduced. The other parameters are Δ = 0, *η* = 0.01, *α* = 3, ${g}_{0}^{g}={g}_{0}^{e}=0.55$, *J* = 20, ${B}_{e,h}={B}_{e,h}^{w}={C}_{h}^{w}=100$, ${C}_{e}^{w}=10$. Reprinted with permission from^81^^©^The Optical Society

To obtain hysteresis, it is necessary to include phase-amplitude coupling between the ES carriers and the GS field, modelled by the *β*-factor in the above. This is similar to the well-known GS only phase-amplitude coupling term *α* arising from the dependence of semiconductor refractive index on carrier density, but it instead couples the GS field to the ES carriers. Such a phase-amplitude coupling arises fundamentally via the semiconductor Bloch equations just as the usual *α*. Thus, even when the ES is not lasing, the changing carrier density still has an impact on the refractive index and on the GS emission. In this way, it also phenomenologically models some aspects of inhomogeneous broadening^81,82^. In the absence of *β*, no hysteresis is obtained. For sufficiently high *β* the hysteresis is found, matching the experiment very well as seen in Fig. 5.

In^83,84^ Lüdge and co-authors consider a microscopic model for the dual system. All of their results are numerical but they consider carefully the experimental results of^81^. They also perform successful analyses for the GS only situation using such a model in^14,85,86^. Rather than having an explicit *α*, they extract the frequency shift of the GS field brought about by changes in the full carrier distribution, thereby going beyond the conventional approach where only the active carrier population is considered (and linear scaling assumed). Because they consider phase amplitude coupling of the GS and all the carriers, there are effective *α*- and *β*-factors in this model, even when inhomogeneous broadening is omitted.

The experimental hysteresis results described above are reproduced successfully for control parameters where the free-running behaviour of the QD laser is emission from the ES only. As with the rate equation model in^81^, the phase-amplitude coupling needs to be sufficiently strong to allow the hysteresis to arise and does not arise for very low values. By changing device and control parameters, they also examine if the bistability can arise for the other free-running operating possibilities: GS only and simultaneous GS and ES lasing. They show that variations on the bistability can in fact also arise in these situations. Further, it turns out that the largest bistable region is found with the GS only lasing parameters. This might seem counter intuitive at first, but can be explained as follows. For optically injected single mode QD lasers operated in the GS only regime, the injected light can cause the intensity of the emitted light to exceed that of the free-running emission but for certain combinations of the injection strength and the detuning, it can also cause it to drop below the free-running value as shown experimentally in^87^ (see Fig. 5.24 therein) and numerically in^83^. When it drops below the free-running emission, it frees up carriers. This can allow the ES threshold to be reached and so emission from the ES can arise.

**Fig. 6:**
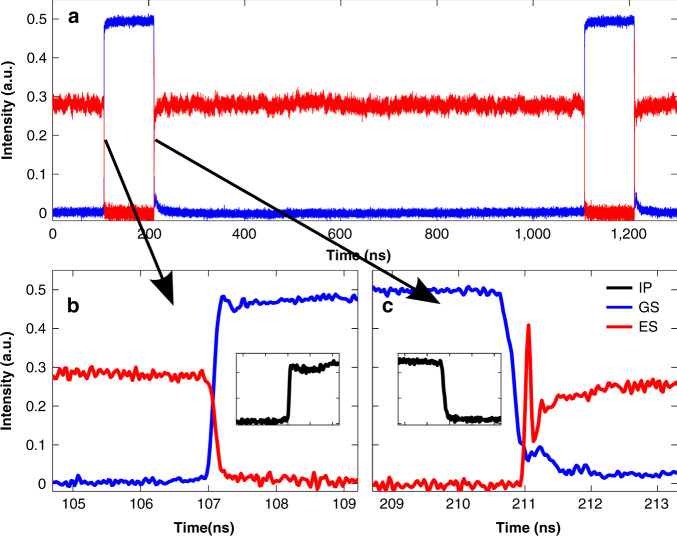
Experimental optical switching between the GS and ES. A Mach-Zehnder modulator is connected to a pulse generator that produces square pulses. The rising and falling edges of an electrical pulse are shown in the insets of (**b**) and (**c**), respectively. During the short squares in (**a**), GS wavelength light is injected into the ES emitting QD laser. The ES quickly turns off and the GS quickly turns on. When the injection drops again (long central plateau in (**a**)) the GS returns to off and the ES switches on again. The injection current of the SL is 84 mA. Note that the coloring of the outputs is reversed compared to the other plots in this paper. Reprinted with permission from^80^^©^The Optical Society

**Fig. 7:**
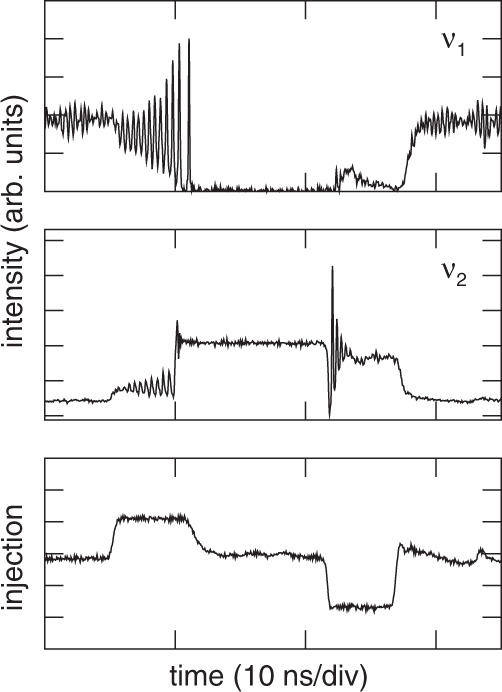
Optically injected multi-quantum well two-colour Fabry-Perot laser. The top panel shows the intensity of the uninjected mode at frequency *ν*_1_; the middle panel shows the injected mode at frequency *ν*_2_; and the bottom panel shows the injected signal. Reprinted with permission from^50^^©^The Optical Society

So far we have discussed only the steady state behaviour of the injection configuration. Dynamics are also of course important. One of the first experiments considering dynamics examined the details of the switching between the CW GS on solution induced by injection and the free-running ES on solution, in^80^. This work thus bridges the gap between the steady state analyses above and the dynamics presented below. In^80^, a similar device to that in^81^ is studied experimentally. Again, it is electrically pumped so as to emit from the ES only when free running. Under continuous wave injection, the ES is fully suppressed and the device lases from the GS only. In order to go beyond steady state results, a Mach Zehnder modulator modulates the injected light to induce switching between the two lasing states. The rise time of the injected GS pulse is ~100 ps. The switching time—the time it takes for the QD laser to switch from ES to GS lasing—is <300 ps, Fig. 6. There is a notable absence of RO oscillations, as is typical for QD devices due to their extremely high RO damping in the GS. The injected pulse has a fall time of ~300 ps. The time to switch back from GS-only lasing to ES-only lasing is <700 ps. In contrast to the GS case, the switching on of the ES is accompanied with one pronounced ring in the intensity suggesting that the RO damping in the ES is high, but not as high as in the GS case. In^50^ a similar scheme is used to switch between two states in a two-colour quantum well based laser. Figure 7 shows results with that system and the very pronounced relaxation oscillation dynamics that ensue. This is in sharp contrast to the almost complete absence of ROs in the QD case. The two state QD switches are extremely fast but likely significantly affected by somewhat slow switching pulses. With injected pulses with shorter rise and fall times, the state switching times would also be shorter.

The optical switching technique is also investigated numerically using the microscopic model in^83^. The QD laser is operated so as to emit from the ES and a square pulse of several ns duration at a wavelength close to that of the GS is injected, with rise and fall times of 100 ps. As the GS square is injected the ES turns off and the GS turns on as in the experiment. The timescales for the GS switch-on of about 300 ps and the GS switch-off of about 900 ps, again, similar to those reported in^80^.

The authors also consider the case where the system is bistable between GS only lasing and dual state emission and apply short perturbations to the injection strength. Beginning with a constant GS injection where the QD laser is phase locked and the emission is from the GS only, a short pulse momentarily decreasing the injection strength can induce a state switch to dual state emission. Conversely, a short pulse momentarily increasing the injection strength can induce a switch back to the ES off state. Similarly to the constant injection switching case, the switch from GS only to dual state lasing is significantly slower than that from dual state to GS only.

We note that in^53^ a number of potential bistable regions where different pulsing dynamics coexist are identified. Such interesting regimes have not been observed experimentally as yet, but may warrant further investigation.

## Boundary dynamics

In conventional optical injection configurations, dynamics are typically found and studied near locking/unlocking boundaries and the dynamical regimes obtained depend on the bifurcations leading to phase locking. Boundary dynamics are also prevalent in the dual state injection case.

### Intrinsic Q switching

**Fig. 8:**
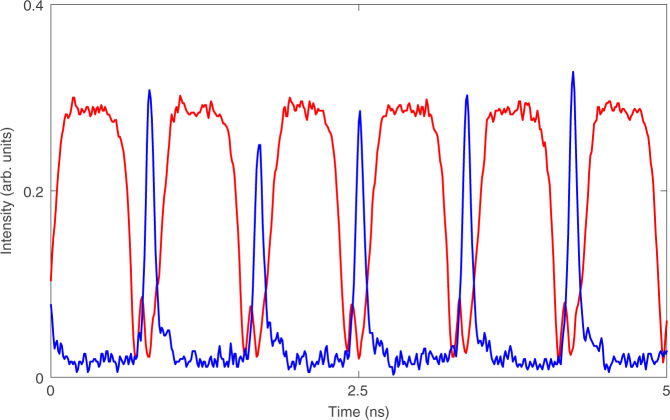
Experimental train of GS dropouts accompanied by sharp ES pulses. Reprinted with permission from^88^^©^The Optical Society

**Fig. 9:**
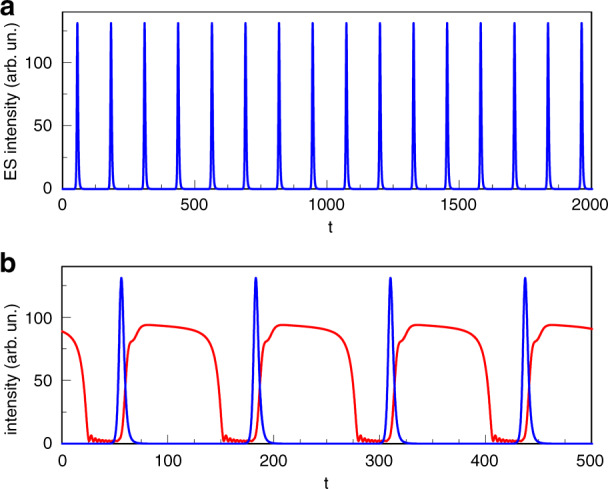
**a** shows a numerically generated train of ES pulses. **b** shows a zoom of (**a**) with the accompanying GS dropouts. The time is scaled by the photon lifetime in the numerics. The small ringing oscillations at the minima of the GS dropouts correspond to oscillations around a saddle focus. For the numerics the parameters are $\varepsilon =3,{{\Delta }}=-0.06,\eta =0.01,\alpha =3,\beta =2.4,{g}_{0}^{g}={g}_{0}^{e}=0.55,J=40,{B}_{e,h}={B}_{e,h}^{w}={C}_{h}^{w}=100,{C}_{e}^{w}=10\ \,{{\mbox{and}}}\,\ {C}_{e}={B}_{e}\,{{\mbox{exp}}}\,\left(-2\right)$. Reprinted with permission from^88^^©^The Optical Society

In^88^, a pulsing regime close to the locking boundary for negative detuning is studied. A fixed injection strength is chosen and the frequency of the injected light is varied. Just outside the unlocking boundary, periodic trains of GS intensity dropouts (with ringing minima) and corresponding sharp antiphase ES pulses of ~80 ps duration are observed. Figs. 8 and 9 show experimental and numerical examples of such periodic traces.

Just inside the locking boundary aperiodic trains are obtained. This finding is qualtiatively identical to that of the conventional locking case, suggesting a limit point/saddle node infinite period (homoclinic) bifurcation (a SNIPER, also known as a saddle node on an invariant cycle - SNIC - bifurcation). We focus on the periodic case here and return to the aperiodic case in Section 3.2.1 below.

The period of the trains can be tuned as shown in^88^ and a tuning range of ~1 GHz (0.3–1.3 GHz) is demonstrated for the injection strength considered. This is much larger than the tuning ranges of passively Q switched semiconductor lasers.

**Fig. 10:**
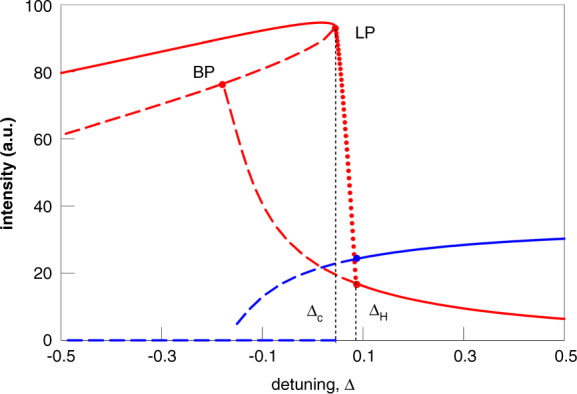
Bifurcation diagram of the GS (red) and ES (blue) intensities vs detuning using the rate equations from^88^. Continuous (dashed) lines correspond to stable (unstable) branches. *LP* denotes a limit point/saddle node bifurcation. This coincides with a homoclinic bifurcation, labelled by Δ_*c*_, forming a SNIPER bifurcation, as with the models in^83^ and^90^. Δ_*H*_ denotes a Hopf bifurcation point. BP denotes an unstable bifurcation point. Reprinted with permission from^88^^©^The Optical Society. Note that the sign of the detuning in this figure is the opposite of that used in the rate equations presented in this review

Using the already described rate equation model, Equations (1) to (5), a bifurcation analysis of the stable solutions is undertaken. Figure 10 shows a mapping of the ES and GS intensities versus the detuning and the corresponding map for the phase of the GS (originally shown in^88^). (Note that in^88^, and thus in Figure 10, the detuning is defined with the opposite sign to that used in this review.) As suspected from the experiment, the negative detuning boundary is defined by a SNIPER bifurcation, just as in the conventional injection case. The position of the limit point/saddle node bifurcation is labelled as LP in Fig. 10. Similar but complementary one parameter bifurcation analyses also revealing the SNIPER boundary are undertaken in^89^ and by Mesaritakis and co-workers in^90^, where in both cases, the varied control parameter is the injection strength. In^83^ and^84^ Meinecke et al. performed a two parameter analysis finding the SNIPER bifurcation and studying its evolution as both the injection strength and the detuning are varied. (Indeed, this figure appears below in this Review as Fig. 19.) Immediately to the right of LP in Fig. 10, periodic trains of GS dropouts and ES pulses are obtained just as in the experiment. Figure 9 shows such a train for Δ = −0.06. Similar to the experiment, the GS dropouts have multiple ringing oscillations (discussed below). The physical explanation of the dropout/pulsing phenomenon is somewhat analogous to the phenomenon of Q switching in lasers with saturable absorbers. After unlocking, the GS intensity drops, and its gain saturates due to Pauli blocking. This saturation of the GS allows carriers to build up in the ES. It acts as a gate allowing the ES carrier population to increase until it eventually overcomes the device losses and yields another sharp ES pulse. The tuning ranges in the injection experiment, however, go well beyond those achieved with Q switching since in that case, the pulse rate is set by the pump and absorber time scales. This intrinsic Q switching phenomenon is unique to QD lasers, arising directly from the carrier cascade pathway described above without any requirement for multisection fabrication.

### Excitability and neuromorphic functionality

#### Stochastic triggering

While periodic trains are found outside the locking boundary, close to the boundary, but inside the locking region, dropout and pulse trains are also observed but with random interpulse/dropout separations.

**Fig. 11:**
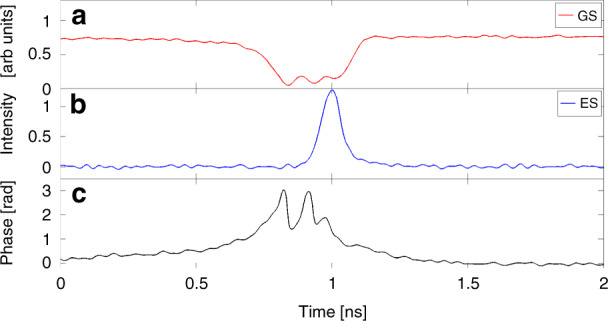
(a) Experimentally observed noise induced GS dropout, (b) the corresponding ES pulse, and (c) the bounded phase trajectory of the GS. **a** Experimentally observed noise induced GS dropout, (**b**) the corresponding ES pulse, and (**c**) the bounded phase trajectory of the GS. Reprinted figure with permission from^89^, M. Dillane, I. Dubinkin, N. Fedorov, T. Erneux, D. Goulding, B. Kelleher, and E.A. Viktorov, Physical Review E 100 012202, 2019. Copyright (2019) by the American Physical Society

**Fig. 12:**
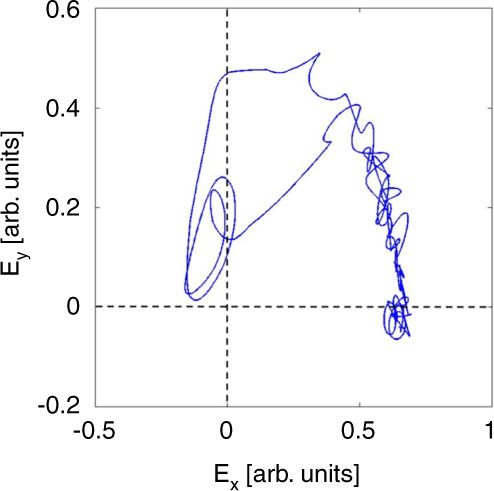
Experimental phasor plot, constructed using an interferometric technique involoving a 3 × 3 coupler^89^. The trajectory shown is that of the GS phasor during one noise induced dropout. The oscillations/loops near the origin indicate the presence of a saddle focus. Reprinted figure with permission from^89^, M. Dillane, I. Dubinkin, N. Fedorov, T. Erneux, D. Goulding, B. Kelleher, and E.A. Viktorov, Physical Review E 100 012202, 2019. Copyright (2019) by the American Physical Society

**Fig. 13:**
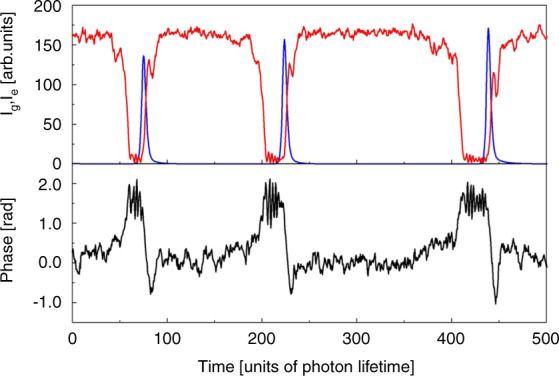
Intensity and phase time traces obtained from numerical simulations. The bounded phase trajectory is clear. The parameters are $\varepsilon =4.44,{{\Delta }}=0,\eta =0.01,\alpha =3,\beta =2.4,{g}_{0}^{g}={g}_{0}^{e}=0.55,J=56,{B}_{e,h}={B}_{e,h}^{w}={C}_{h}^{w}=100,{C}_{e}^{w}=10\ \,{{\mbox{and}}}\,\ {C}_{e}={B}_{e}\,{{\mbox{exp}}}\,\left(-2\right)$. The noise term *D* = 0.25. Reprinted figure with permission from^89^, M. Dillane, I. Dubinkin, N. Fedorov, T. Erneux, D. Goulding, B. Kelleher, and E.A. Viktorov, Physical Review E 100 012202, 2019. Copyright (2019) by the American Physical Society

These aperiodic trains are further investigated in^89^. Such pulsing regimes in the vicinity of SNIPER bifurcations can be utilised in photonic neural networks and are known as Type I excitable pulses^91^. One of the characteristic features of excitability is the existence of a perturbation threshold. To trigger an excitation, a perturbation greater than this threshold is required. In^89^, noise is the perturbing element, so individual pulses fire with random interpulse intervals. The evolution of the phase of the GS during a dropout is investigated using a 3 × 3 interferometric technique^89,92^ and is shown in Fig. 11. The phasor representation of the GS field is shown in Fig. 12. The observed bounded phase rotation contrasts with the 2*π* phase slips observed at the locking boundaries of optically injected single colour QD lasers and indeed, optical injection of semiconductor lasers in general^17^. Bounded phase phenomena are well known in the injection configuration but are typically associated with Hopf bifurcations^92,93^, rather than the SNIPER found here. Noise is included in the rate equation model via the addition of a stochastic term $\sqrt{D}\xi \left(t\right)$ to the right hand side of Equation (1), where $\xi \left(t\right)$ is a unit Gaussian white noise term and *D* is a constant. This results in noise-induced excitable pulses just as in the experiment as shown in Fig. 13. The trajectory of an individual pulse begins at the fixed, phase-locked, point. Noise then perturbs the system past a separatrix and the excitable trajectory begins. Physically, the GS begins to turn off and there is an intensity dropout. At the minimum of the dropout there are multiple ringing oscillations due to a saddle focus. Once the system hits this focus the ES emits its short, sharp pulse, and the GS returns to the phase-locked state. The bounded phase trajectory further emphasises the underlying physical differences between the two systems and the non-Adler nature of the dual state case. We return to this below when discussing reports on deterministic triggering of dual state excitability Fig. 12.

#### Deterministic triggering

One of the most exciting recent developments in laser dynamics is the pursuit of neuromorphic configurations with a view to novel data and information processing. See for example^94^ and references therein. Biological neurons interact and process data via trains of excitable pulses. The excitable responses of optically injected semiconductor lasers are analogous but are many orders of magnitude faster. This speed, allied with the ubiquity, ease of control, and compact size of semiconductor lasers, has led to intense efforts to employ them as artificial neurons. QD lasers have started to feature prominently in such studies. In the GS only regime, several excitable regimes have been identified and studied^17,20,21^. However in this review, we wish to discuss efforts that utilise dual state mechanisms.

One of the principal requirements for using excitable pulsing for applications is deterministic control of the pulses. The prototypical demonstration for Type I excitability in the laser system is to quickly perturb the phase and show the existence of a threshold perturbation strength leading to pulses. For conventional, single state excitability, this has been shown in^18,95,96^. A threshold can also be found for incoherent perturbations as shown in^97^.

**Fig. 14:**
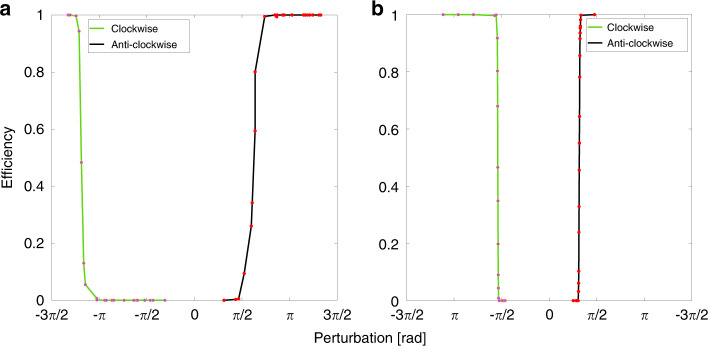
**a** Experimental efficiency curves for triggering excitable GS dropouts/ES pulses. Both clockwise and anticlockwise perturbations exhibit distinct thresholds. **b** Numerical efficiency curves, again clearly displaying distinct thresholds for clockise and anticlockwise directions. Reprinted with permission from^32^^©^The Optical Society

In^32^ it is shown that one can also deterministically trigger the excitations in the dual state system. The system is placed as close as possible to the negative detuning boundary but far enough away so that no excitations are stochastically generated. Fast phase perturbations are then applied using a phase modulator via the rising and falling edges of electrical pulses sent from a pulse generator. These are referred to as clockwise and anticlockwise in the phasor sense. For anticlockwise perturbations the relative phase of the secondary laser is quickly increased and vice versa for clockwise perturbations. Beginning with anticlockwise perturbations, Fig. 14 shows the efficiency of pulse excitation as a function of the perturbation strength and a clear threshold is obtained as in the conventional case.

**Fig. 15:**
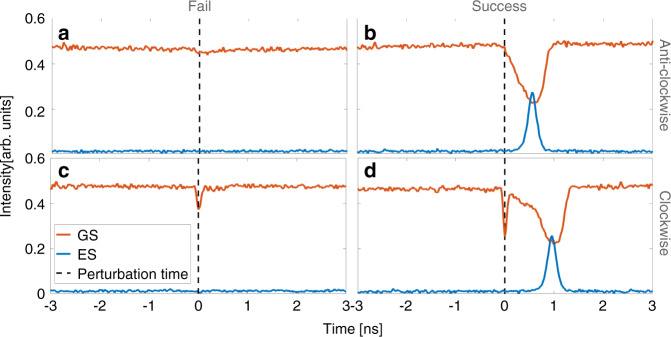
(a) and (b) show the responses of the system to anticlockwise perturbations with magnitudes of 1 and 4.2 rad, respectively. **a**, **b** Show the responses of the system to anticlockwise perturbations with magnitudes of 1 and 4.2 rad, respectively. **c**, **d** Show the responses when clockwise perturbations of −3 and −4.2 rad, respectively, are applied. The ES response vanishes for the below threshold perturbations. Reprinted with permission from^32^^©^The Optical Society

Excitability is often described as an all or nothing phenomenon: below threshold perturbations do not yield pulses and above threshold perturbations do. However, in reality, there are always effects from the below threshold perturbations that are visible, but they are simply much less prominent than full pulse excitations. This remains true for the GS in this case, but because of the dual state nature of the excitability in the system, the excitable ES pulses are truly all or nothing as shown in Fig. 15. Since the ES pulse only arises at the end of the GS dropout, the ES response to below threshold perturbations vanishes as seen in Fig. 15 (a) and (c) while a full pulse is obtained in Fig. 15 (b) and (d).

An extremely novel feature is found when clockwise perturbations are applied. A threshold for excitation is also found in the clockwise case. In the conventional case, clockwise perturbations do not yield excitability. There is no threshold and instead there is a continuous evolution of the response to clockwise perturbations. This is because the underlying structure is that of the Adler mechanism with phase playing the central role. In the dual state system, this is no longer the case. The excitability reported is manifestly not of Adler form; indeed, the phase is bounded as already described, in direct contrast to the unbounded trajectories of the Adler model. Further, the excitable trajectory can be physically understood as a switching off of the GS in the dropout, and a subsequent return to GS lasing rather than a momentary “beating” between the primary and secondary lasers.

The threshold is greater in the clockwise direction than in the anticlockwise case and so there are asymmetric thresholds for excitability in this system. This leads to an intriguing scenario. As with conventional type I excitability, one can integrate several sub threshold perturbations to excite a pulse, achieving an integrate and fire artificial neuron. However, due to the double threshold and the 2*π* periodicity of the phase, a perturbation can be too large to result in an excitation. Thus, one can integrate and inhibit if the perturbations are too large. Further, one can use the two directions to create both excitatory and inhibitory pulses. Thus, the system offers tremendous flexibility in its neuromorphic operation.

In^90^ Mesaritakis and co-workers consider a numerical analysis of a single section QD laser, also with a view to integrate and fire functionality. They ultilize a multi-population model, with electron-hole dynamics describing waveband transitions from both the ground and excited energy state. The electric fields of both the GS and ES are found and the carriers in the bulk semiconductor layer are included. They focus on the behaviour near the SNIPER (SNIC) bifurcation as also previously reported in^83,88,89^ finding pulse/dropout trains similar to those described above and first reported in^88^. The emergence of this phenomenon thus appears to be generic to all models. The authors also perform a numerical analysis of the effect of periodic perturbations of the injection strength on ES pulse and GS dropout generation and map the effect of perturbation strength and period. The underlying physics is very reminiscent of the work of Hurtado and Javaloyes on optically injected VCSELs in^46^, where temporary reductions in the injection strength produced periodic pulse trains. Good control of the repetition rate is suggested by the numerics in^90^. That this should be so is also strongly suggested by the demonstration of repetition rate tuning in^88^ where rather than varying the strength of the primary laser, the frequency is changed.

In^73^, Mesaritakis et al. also analysed a two section mode locked QD laser injected by an identical two section device. Both excitatory and inhibitory pulses can be excited depending on the injected signal and the control parameters of the neuron device (the injected laser). They show that they can achieve either excitation or inhibition simply by changing the reverse bias of the absorber section.

### Two colour bursting

**Fig. 16:**
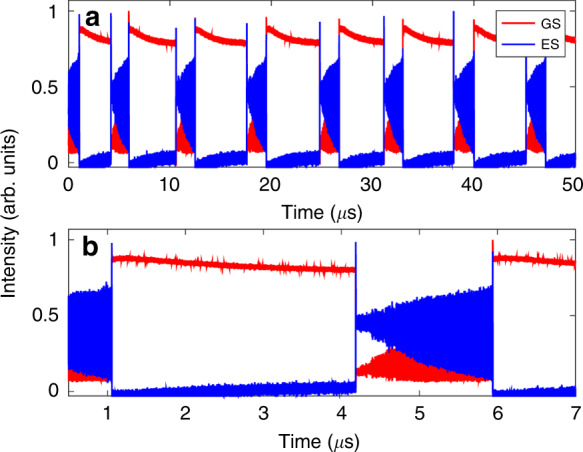
Periodic switching between a quiet state and a bursting state. During the quiet state GS light is the dominant output while during the bursting state the ES is dominant. Reprinted figure from^22^

**Fig. 17:**
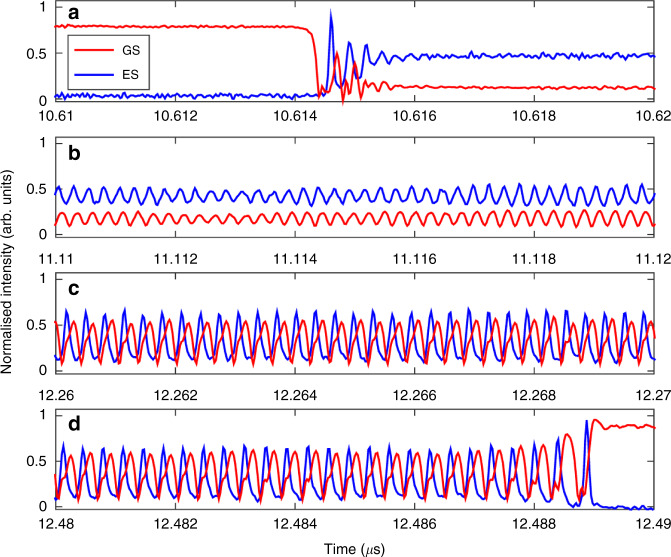
Zoom of an individual bursting interval. The intensities of the states oscillate in antiphase, and the amplitude and the frequency of the oscillations continuously grow until there is a state switch back to the quiet state. An optothermal coupling is responsible for an intrinsic detuning sweep and subsequent slow passage through bifurcation points leading to the observed outputs. Reprinted figures from^22^

**Fig. 18:**
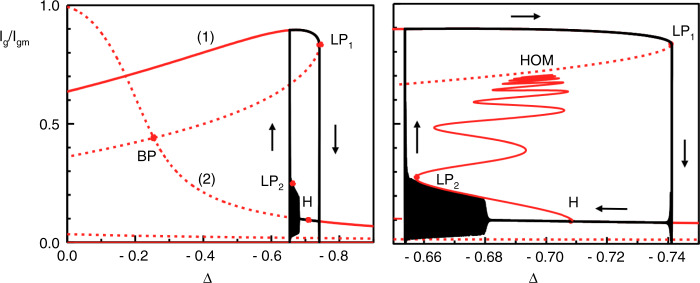
The left panel shows a bifurcation diagram for the intensity of the GS as the detuning is changed, where the optothermal coupling is not included. Stable and unstable branches are shown by full and dotted red lines, respectively. The points LP_1_ and H mark a limit point of steady states and a Hopf bifurcation point, respectively. Including the optothermal effect, the black, deterministic bursting cycle is found. The right panel shows a zoom of bursting region and also some extra details. In particular, a branch of periodic solutions (represented by their maximum intensities) emerges from H and snakes with various stability changes until it reaches the unstable steady state branch at a homoclinic bifurcation point (HOM). The figure shows that the bursting arises from a slow passage through H, and then follows the stable branch of periodic solutions until it reaches a limit-point of limit-cycles (LP_2_). Reprinted figure from^22^. Note that the sign of the detuning in this figure is the opposite to that used in the rate equations presented in this review

In the GS only system, at high injection strengths, an optothermal coupling induced square wave excitability is reported in^21^ and controlled triggering of these pulses is demonstrated^18^. The phenomenon results from the optothermal destruction of a phase-locked bistability. As shown in^18^ there are two potential routes to the square waves, both via a new Hopf bifurcation; one leads to a canard explosion and the other to a subcritical Hopf induced bistability. There is a similar dynamic regime in the dual state configuration when the injection strength is increased beyond the excitable dropout/pulse region. In^22^, an optothermally induced periodic square wave switching on microsecond time scales appears, associated with the two states from a dual state bistability found in the bare, no optothermal coupling, system. There is a periodic switching between a quiescent interval, where the GS emits a constant intensity and the ES is off, and an active interval distinguished by complex oscillations as shown in Fig. 16. In fact, there are multiple distinct regions within the active interval as shown in Fig. 17. The initial GS dropout and ES turn on is characterised by a few damped oscillations in both states followed by a prolonged almost flat region of low GS intensity and higher ES intensity. After a certain critical time, ever growing oscillations appear in both outputs. These oscillations last for a significant duration (in^22^ a bursting duration of 1.5 μs is shown). Following the oscillations the GS returns to its initial quiescent state. The fast-slow time scales and the alternation between large and small amplitude oscillations suggest that these are mixed mode oscillations (MMOs)^98^. In^22^ the rate equation model is supplemented with an optothermal coupling that allows the detuning to vary with the total intensity. This reproduces the dynamics and also explains the bursting cycle. The entire cycle is manifested from a series of slow passages through bifurcation points as shown in Fig. 18. The initial dropout from the quiescent phase arises from a slow passage through the limit point LP_1_. The transit from constant intensities to the evolving oscillations arises via a slow passage through the Hopf bifurcation H. Finally, the return to the quiescent phase arises via a slow passage through a limit point of limit cycles LP_2_. (Note that in^22^, and thus in Fig. 18, the detuning is defined with the opposite sign to that used in this review.)

Over the course of a cycle, effects such as changing carrier recombination levels result in temperature changes in the device, in turn changing the frequency of the device, yielding a deterministic sweep of the detuning. This is similar to the Type II excitability phenomenon reported in^21^. A snaking branch of periodic solutions emerges from a Hopf bifurcation, H. The period and intensity of the bursting oscillations match perfectly those of the first branch, as seen in the right hand panel of Fig. 18. The duration of the bursting phase can be from times of approximately 1 μs to as long as several 10s of μs, via adjustments of the injection strength and the detuning. This could provide useful applications in both artificial neural networks and in optical communications since bursts can contain more information than single pulses.

As with many of the phenomena described here, this is again a result that has its basis in the existence of an inherent bistability. The bistability is broken by the thermal coupling yielding the fast-slow MMO regime. MMOs are of course a generalised canard phenomenon^98^ and so this is yet another instance of the dual state behaviour mimicking the single state behaviour but with added richness in the structure.

### Device (in)dependence

We stress here that all of the dynamic features reviewed here, including the dropouts and the bursting phenomenon, have been investigated and observed with many different devices of varied lengths, threshold currents, and losses. The phenomena are extremely robust and generic. Further, optothermal effects appear with multiple different mounting systems and even in the absence of temperature control.

As already discussed, two particular but generic properties of QD lasers are crucial for much of the dynamics described in this work. Namely, the high RO damping and the dual state lasing. Depending on the specific dynamic feature either or both of these can be central. For example, antiphase excitability is plausible for other dual state systems, while the appearance of a phase locked bistability (broken or otherwise) requires the high RO damping.

## Where is the chaos?

**Fig. 19:**
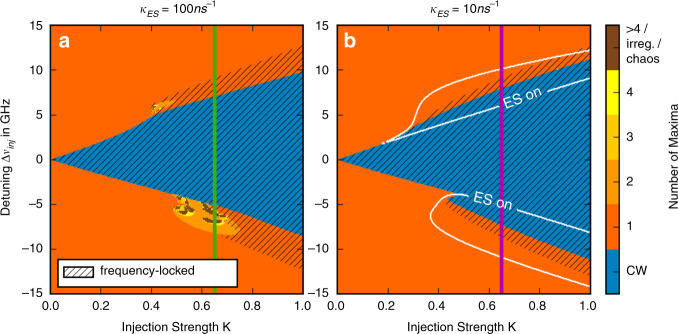
Maps comparing a single-colour laser (**a**) and a two-state laser (**b**) under optical injection using the microscopic model. There are regions of chaos near the unlocking boundaries of of the single-colour laser map. However, in the two-state case, only phase locked and period one dynamics are found, with no chaos or even higher period dynamics observed in the map. Reprinted with permission from^84^, S. Meinecke, B. Lingnau and K. Lüdge, in Physics and Simulation of Optoelectronic Devices XXV, Vol. 10098, International Society for Optics and Photonics (SPIE, 2017)

With all of the dynamic scenarios described above, one feature is conspicuous by its absence: chaos. Chaotic operation is typically one of the standout features of optically injected semiconductor lasers. A conventional semiconductor laser can be well described using just two equations. The inclusion of optical injection increases the dimensionality of the system and allows the generation of chaos^99^. With conventional semiconductor material, prominent areas of chaos are found, in large part due to the weak damping of the ROs. However, even with highly damped QD lasers, chaos is found under injection albeit with a smaller footprint in the mapping^16,83,84^. When the laser is biased so that the free-running operation is from the ES only, chaos does not seem to appear in the injection map at all, or if it does, it does so in extremely small regions. The system has a high dimensionality, higher than the GS only system where chaos does arise. However, as shown in^83,84^—see Fig. 19—and in agreement with the predictions of the rate equation model in Equations (1)-(5), chaos is simply not a feature of the dual state injection system. Rather, regions where chaos would appear in the single state system become dual emission states. This is borne out by experiment.

Of course, the absence of chaos does not equate to an absence of complexity. There is ample complexity in the interplay between the states. Even in the free-running case, the quenching of the GS due to ES lasing is a very complex phenomenon, while the optothermal bursting cycle described above is incredibly rich in structure with MMOs, homoclinic snaking and slow passage phenomena.

## Outlook: photonic integration

The maturation of integration technology presents great opportunities for applications based on coupled semiconductor lasers. Several aspects of QD lasers make these extremely attractive for implementation on photonic integrated circuits. The high RO damping means that they may be coupled on chip without the requirement for isolators, they display superb thermal insensitivity, and they offer a multitude of controllable dynamic responses from coherent outputs to deterministic dual state bursting. Further, the carrier confinement in QD material results in a much higher tolerance to III-V/Si crystalline defects compared to quantum well based material^33^, strongly encouraging further research on the integration of QD lasers (and indeed other components based on QD material) on Si. What’s more, since the ES lasing regime is utilised, it should be possible to integrate very short lasers, as can be done with conventional quantum well based devices. Thus, the integration of QD lasers is extremely promising with a very appealing, relative simplicity.

In a recent work^100^, on-chip coupled quantum well based devices are analysed and their potential as building blocks for excitable neuromorphic networks highlighted. Utilising QD material in place of the quantum well material would potentially enhance the capabilities in this direction, and bring a multitude of controllable, excitable regimes and in particular both the dual state excitability and dual state bursting MMOs described above.

The stability of phase-locked QD lasers means conventional information processing applications could also benefit. In particular, the intrinsic bistabilities and consequent memory and optical flip flop functionalities of dual state QD lasers are extremely encouraging for optical signal processing tools, including optical logic gates and interconnects.
